# Failure of malaria chemoprophylaxis with mefloquine in an oversize traveller to Mozambique

**DOI:** 10.1186/1475-2875-12-451

**Published:** 2013-12-18

**Authors:** Federico Gobbi, Andrea Rossanese, Dora Buonfrate, Andrea Angheben, Gianluigi Lunardi, Zeno Bisoffi

**Affiliations:** 1 Centre for Tropical Diseases, Sacro Cuore-Don Calabria Hospital, Verona, Negrar, Italy; 2 Oncology Department, Sacro Cuore-Don Calabria Hospital, Verona, Negrar, Italy

**Keywords:** Failure, Mefloquine, Chemoprophylaxis, Malaria

## Abstract

A case of failure of mefloquine prophylaxis in an oversize traveller, who correctly took the drug. This case seems to be attributed to mefloquine resistance, however it is suggested that mefloquine dosage should be modulated by body weight, as is already indicated by some authorities.

## Background

The correct dosage for mefloquine in anti-malarial chemoprophylaxis is 5 mg/kg [1]. The vast majority of international [2,3] and national [4] guidelines on malaria prevention suggests a prophylactic dose of 250 mg per week in adult travellers. However, regardless the product label, some national Travel Medicine Societies, such as the Swiss and the Austrian ones, suggest to modulate the drug dosage by body weight (Table 1) [5,6]: 1 ½ tablet per week for patients weighing > 90 kg, two tablets per week for the patients weighing > 120 kg. The dosage issue concerns not only the overweight, but also the low weight travellers. Those weighing < 60 kg may poorly tolerate a whole tablet of mefloquine per week [7-9].

**Table 1:** Dosage of mefloquine tablet (250 mg) in Swiss/Austrian malaria prophylaxis recommendations

**> 120 kg**	**2 tablets (twice weekly on tablet: e.g. Mon & Thu)**
> 90 kg	1 and ½ tablets (twice weekly on tablet: e.g. Mon & Thu)
> 60 kg	1 tablet
40-60 kg	¾ tablet
20-40 kg	½ tablet
10-20 kg	¼ tablet
< 10 kg	5 mg /kg

A case is presented of mefloquine failure in an oversize traveller, that was initially attributed to insufficient dosage.

### Case presentation

A 50-year-old Italian patient was admitted to the Centre for Tropical Diseases (CTD) with fever (39°C) and diarrhoea since three days. He reported having visited many Catholic missions over several years (he is a priest) in sub-Saharan Africa, Asia and Latin America. The average length of stay was between 10 and 14 days. He had been vaccinated against yellow fever, hepatitis A and B, and typhoid fever. From 20^th^ to 31^st^ March 2012, he had travelled to Mozambique, regularly taking malaria chemoprophylaxis with mefloquine, one tablet (250 mg) weekly, starting one week before travelling and continuing for four weeks after return: he took the last tablet on 24^th^ April. He presented to CTD on May 18^th^ 2012. His weight was 144 kg. He had no history of alcohol, tobacco or illicit drugs abuse. He was not under any long-term medication. The hearth rate was 90 beats per minute and the blood pressure 140/70 mm Hg. Physical examination was unremarkable, except for a distended abdomen. Quantitative buffy coat (QBC), antigen malarial test and blood smears resulted positive for *Plasmodium falciparum* malaria, with a parasitaemia of 0.13% (6330 /μl). The blood tests showed 7430 WBC/μl, C reactive protein 142 mg/L. The patient was treated with artemether (tablet 50 mg), eight tablets the first day, then three tablets/day until the fifth day, followed by four tablets of sulphamethoxypyrazine/pyrimethamine 500 mg/25 mg (artemisinin-based combination artemisinin-based combination therapies were not yet available). After two days of treatment, blood films resulted negative and remained so at follow-up, one month later.

Whole blood and plasma mefloquine concentrations were retrospectively evaluated on cryo- preserved (-80°C) samples taken on admission (25 days after the last mefloquine intake), by high pressure liquid chromatography (HPLC) coupled with ultraviolet diode-array detection [10,11].

Whole blood and plasma mefloquine concentrations were 516 and 320 ng/mL, respectively.

## Discussion

Despite a correct anti-malarial chemoprophylaxis, the patient developed *P. falciparum* malaria. Therefore, it was concluded that either the strain of *P. falciparum* was resistant to mefloquine, or its dose was insufficient according to the patient’s body weight. At first, the failure was attributed to an insufficient dosage according to the patient’s weight (144 kg), but later mefloquine concentration was assessed on pre-treatment plasma and blood samples that had been stored.

The mefloquine level in the patient’s bloodstream at admission was within the expected range, considering the breakthrough mefloquine levels >620 ng/ml and a half-life of 21 days (Product information Lariam®, Roche, the Netherlands). Considering these data, *P. falciparum* resistance to mefloquine was more probable, despite the limitation of a single point in time determination.

A review of literature was performed to collect the characteristics and probable causes of reported cases of failure of mefloquine prophylaxis. PubMed was searched using the following keywords: mefloquine, chemoprophylaxis, failure, Africa. Thirteen papers were retrieved [12-24], describing a total of 31 cases (Table 2). Only three cases were reported in the last 15 years, while the others were reported between 1990 and 1998. In most of the cases (25/31) the infection had been acquired in West Africa, in six cases in East Africa (one each in Malawi, Tanzania, Madagascar, Mozambique, and two in Somalia). Six patients were females and 25 males; six were children and 25 were adults between 18 and 50 years of age. Three patients developed *P. falciparum* malaria while in the endemic country, 28 patients after return. In the latter group, 16 travellers became ill within four weeks after return (still under prophylaxis) and 12 developed the disease after the last dose of mefloquine. The median onset of symptoms after return was 28 days (range 3–91 days). In all cases resistance to mefloquine was presumed, although for some of them drug concentration in plasma was not available.

**Table 2:** Papers describing mefloquine prophylaxis failure in travellers to Africa

**Author**	**Age**	**Sex**	**Country of infection**	**Symptoms onset (before return)**	**Symptoms onset (days after return)**
Callen (2006)	25	M	Gambia	-	4
Wichmann (2003)	44	F	Tanzania	-	23
Gary-Toussaint (2002)	45	M	Senegal	-	41
Gizolme (1998)	40, 12, 9	F, M, F	Togo	-	63, 77, 91
Lobel (1998)	31, 22, 30, 26, 25	M, M, M, F, M	Niger, Sierra Leone, Cameroon, RCA	Y	-
Matteelli (1995)	20	M	Mozambique	-	3
Magill (1993)	23	M	Somalia	Y	-
	21	M	Somalia	Y	-
Raccurt (1991)	3, 30, 28, 22, 40, 44, 23, 23, 27	F, M, M, M, M, M, M, M, M	Senegal, Burkina Faso, Niger, Nigeria, Mali, Ivory Coast, Togo	-	3, 4, 6, 13, 15, 19, 21, 25, 26
Ringwald (1990)	5, 28, 34, 37	M, M, M, M,	Burkina Faso, RCA, Madagascar, Togo	-	19, 26, 31, 32
Ooi (1991)	26	M	Malawi, Kenya	-	21
Durieux (1990)	18	M	Burkina Faso	-	58
Gay (1990)	6	M	Senegal, Ivory coast	-	55
Bricaire (1990)	6, 34, 40, 45, 50	M, M, M, M, F	Mali, Sierra Leone, Senegal, RCA	-	7, 30, 45, 46, 61

In relation to the first suspicion, there is no agreement on the need to adjust mefloquine regimen to body weight. It is worth considering this factor, though, also taking into account the lipophilic behaviour of this drug, which has an apparent volume of distribution between 13 to 40 1 kg^-1^ with a mean of 20 1 kg^-1^. Both apparent volume of distribution and individual systemic clearance are probably influenced by body fat, thus causing different pharmacokinetic profiles [7,25,26].

In conclusion, although in this case the failure of mefloquine prophylaxis was most probably due to drug resistance, the indication given by some scientific societies that mefloquine dosage should be modulated by body weight seems reasonable and should probably be considered in new guidelines.

### Consent

Written informed consent was obtained from the patient for the publication of this report.

## Competing interests

The authors declare that they have no competing interests.

## Authors’ contribution

FG, AR visited and followed-up the patient. AA and DB performed Pubmed search. FG and DB have been involved in drafting the manuscript. GL detected drug concentration. ZB and AA revised the manuscript critically for important intellectual content. All the authors have given final approval of the version to be published.

## References

[B1] Schlagenhauf P Adamcova M Regep L Schaerer MT Bansod S Rhein HG Use of mefloquine in children - a review of dosage, pharmacokinetics and tolerability data MalarJ 2011 12 292 10.1186/1475-2875-10-292 21981927 PMC3215676

[B2] CDC Health Information for International Travel 2012 The yellow book http://www.cdc.gov/yellowbook 10.4269/ajtmh.16-0627 PMC5094242 30851020

[B3] WHO International travel and health 2012 Geneva: World Health Organization http://www.who.int/ith

[B4] Lalloo DG Shingadia D Pasvol G Chiodini PL Whitty CJ Beeching NJ Hill DR Warrell DA Bannister BA UK malaria treatment guidelines J Infection 2007 12 111 121 10.1016/j.jinf.2006.12.003 17215045

[B5] DTG Empfehlungen zur Malaravorbeugung 2011 http://www.dtg.org

[B6] Hatz C Nothdurft H Malaria protection for short-term travelers Internist 2006 12 8 810 812 814–817 10.1007/s00108-006-1653-4 16845539

[B7] van Riemsdijk MM Sturkenboom MC Ditters JM Tulen JH Ligthelm RJ Overbosch D Stricker BH Low body mass index is associated with an increased risk of neuropsychiatric adverse events and concentration impairment in women on mefloquine Br J Clin Pharmacol 2004 12 506 512 10.1046/j.1365-2125.2003.02035.x 15025750 PMC1884471

[B8] Schlagenhauf P Tschopp A Johnson R Nothdurft HD Beck B Schwartz E Herold M Krebs B Veit O Allwinn R Steffen R Tolerability of malaria chemoprophylaxis in non-immune travellers to sub-Saharan Africa: multicentre, randomised, double blind, four arm study BMJ 2003 12 1078 10.1136/bmj.327.7423.1078 14604928 PMC261741

[B9] Ollivier L Tifratene K Josse R Keundjian A Boutin JP The relationship between body weight and tolerance to mefloquine prophylaxis in non-immune adults: results of a questionnaire-based study Ann Trop Med Parasitol 2004 12 639 641 10.1179/000349804225021262 15324471

[B10] Green MD Bergqvist Y Mount DL Corbett S D'Souza MJ Improved validated assay for the determination of mefloquine and its carboxy metabolite in plasma, serum and whole blood using solid-phase extraction and high-performance liquid chromatography J Chromatogr B Biomed Sci Appl 1999 12 159 165 10.1016/S0378-4347(99)00080-8 10360435

[B11] Gutman J Green M Durand S Rojas OV Ganguly B Quezada WM Utz GC Slutsker L Ruebush TK 2nd Bacon DJ Mefloquine pharmacokinetics and mefloquine-artesunate effectiveness in Peruvian patients with uncomplicated *Plasmodium falciparum* malaria Malar J 2009 12 58 10.1186/1475-2875-8-58 19358697 PMC2674465

[B12] Callen EC Church CO *Plasmodium falciparum* malaria associated with mefloquine failure in Gambia Pharmacotherapy 2006 12 1526 1528 10.1592/phco.26.10.1526 16999663

[B13] Wichmann O Betschart B Loscher T Nothdurft HD Sonnenburg FV Jelinek T Prophylaxis failure due to probable mefloquine resistant *P. falciparum* from Tanzania Acta Trop 2003 12 63 65 10.1016/S0001-706X(03)00003-2 12711104

[B14] Gari-Toussaint M Pradines B Mondain V Keundjian A Dellamonica P Le Fichoux Y [Senegal and malaria. True prophylactic failure of mefloquine] (in French) Presse Med 2002 12 1136 12162100

[B15] Gizolme D Receveur MC Bouzidi P Theron P [Relative failure of mefloquine chemoprophylaxis on the occasion of a trip to Togo. 3 cases within the same family] (in French) Presse Med 1998 12 1575 1576 9819587

[B16] Lobel HO Varma JK Miani M Green M Todd GD Grady K Barber AM Monitoring for mefloquine-resistant *Plasmodium falciparum* in Africa: implications for travelers’ health Am J Trop Med Hyg 1998 12 129 132 9684640 10.4269/ajtmh.1998.59.129

[B17] Matteelli A Chiodera A Castelli F Caligaris S Minardi C Carosi G Failure of mefloquine chemoprophylaxis for malaria in Mozambique J Travel Med 1995 12 260 261 10.1111/j.1708-8305.1995.tb00673.x 9815405

[B18] Magill AJ Smoak BL Failure of mefloquine chemoprophylaxis for malaria in Somalia N Engl J Med 1993 12 1206 8377800 10.1056/NEJM199310143291617

[B19] Raccurt CP Dumestre-Toulet V Abraham E Le Bras M Brachet-Liermain A Ripert C Failure of falciparum malaria prophylaxis by mefloquine in travelers from West Africa Am J Trop Med Hyg 1991 12 319 324 1928565 10.4269/ajtmh.1991.45.319

[B20] Ooi WW Failure of mefloquine prophylaxis in east Africa N Engl J Med 1991 12 2 130 1984183 10.1056/NEJM199101103240216

[B21] Durieux I Boibieux A Biron F Ringwald P Piens MA Peyramond D [Failure of chemoprevention with mefloquine in western Africa] (in French) Presse Med 1990 12 33 1548 1549 2146671

[B22] Ringwald P Bartczak S Le Bras J Bricaire F Matheron S Bauchet J Coulaud JP Failure of anti-malarial prophylaxis with mefloquine in Africa Trans R Soc Trop Med Hyg 1990 12 348 349 10.1016/0035-9203(90)90311-2 2260164

[B23] Gay F Binet MH Bustos MD Rouveix B Danis M Roy C Gentilini M Mefloquine failure in child contracting falciparum malaria in West Africa Lancet 1990 12 120 121 10.1016/0140-6736(90)90597-X 1967412

[B24] Bricaire F Gay F Caumes E Datry A Bustos D Felix H Paris L Danis M Gentilini M [Failure of prevention of malaria by mefloquine in West Africa] (in French) Ann Med Onterne 1990 12 6 512 514 2285203

[B25] Karbwang J White NJ Clinical pharmacokinetics of mefloquine Clin Pharmacokinet 1990 12 264 279 10.2165/00003088-199019040-00002 2208897

[B26] Palmer KJ Holliday SM Brogden RN Mefloquine: a review of its antimalarial activity, pharmacokinetic properties and therapeutic efficacy Drugs 1993 12 430 475 7682911 10.2165/00003495-199345030-00009

